# Sustainability principle for the ethics of healthcare resource allocation

**DOI:** 10.1136/medethics-2020-106644

**Published:** 2020-11-05

**Authors:** Christian Munthe, Davide Fumagalli, Erik Malmqvist

**Affiliations:** 1 Department of Philosophy, Linguistics and Theory of Science, University of Gothenburg, Goteborg, Sweden; 2 Centre for Antibiotic Resistance Research (CARe), University of Gothenburg, Goteborg, Sweden

**Keywords:** allocation of health care resources, environmental ethics, health care economics, public policy, resource allocation

## Abstract

We propose a principle of sustainability to complement established principles used for justifying healthcare resource allocation. We argue that the application of established principles of equal treatment, need, prognosis and cost-effectiveness gives rise to what we call negative dynamics: a gradual depletion of the value possible to generate through healthcare. These principles should therefore be complemented by a sustainability principle, making the prospect of negative dynamics a further factor to consider, and possibly outweigh considerations highlighted by the other principles. We demonstrate how this principle may take different forms, and show that a commitment to sustainability is supported by considerations internal to the ethical principles already guiding healthcare resource allocation. We also consider two objections. The first of these, we argue, is either based on implausible assumptions or begs the question, whereas the second can be adequately accommodated by the principle we propose.

## Introduction

We propose a principle of sustainability to complement established normative principles used for justifying resource allocation in healthcare. We use three distinct cases to illustrate the need for such a complementary principle, one regarding the cost dynamics of drug procurement, one regarding the environmental dimension of antibiotic resistance and one regarding drug shortages. We start by mapping a general structure for how the normative justification of resource allocation decisions is commonly institutionalised, and use this model to formulate a general argument for sustainability as a core concern in the justification of healthcare resource allocation. The three cases are brought in to illustrate how such a principle is essential to counteract what we term *negative dynamics* that would undermine values backing up the other normative considerations used to justify healthcare resource allocation. We end by considering some basic ethical perspectives that may be used to question the addition of a sustainability principle, arguing that these are either unable to support the other normative considerations used for healthcare resource allocation, or, if adjusted to accommodate these considerations, compatible with a sustainability principle.

## Justification of resource allocation decisions in healthcare

All healthcare systems need to allocate scarce resources, and the justification of such allocation needs to be based on normative principles. Drawing on general moral and political philosophy, ethicists have debated what such normative principles may be justified and how. Nevertheless, in practice, the operational norms that guide actual decision-making in this area tend to be similar across health systems,^1^ and usually include the following principles:

- Need: greater need of care justifies allocating more resources to help the patient group in question (and vice versa).
- Prognosis: greater expected health effect of an intervention justifies allocating more resources to this intervention (and vice versa).^1^
- Equal treatment: equal claims based on need and prognosis justify equal priority for resource allocation (including the balancing of benefits against costs).
- Cost-effectiveness: meeting a prioritised need with a prioritised intervention should not spend more resources than necessary.^2^

There are well-known disagreements on and differences between countries and regions regarding how these principles are operationalised, and how they should be balanced or traded off in cases of conflict. For instance, while the Swedish regulation on healthcare priority setting is written to exclude instrumental effects on other aspects than the individual patient’s health or collective evaluation of the need and prognosis aspects, the UK system for priority setting and health economic evaluation allows wider room for collectivist or economic arguments for recommended resource allocation, for example, that a condition is widespread. In addition, although these principles express a widely recognised ideal, it is also well known that the *realpolitik* of healthcare resource allocation often results in having certain groups of patients win the attention of decision-makers on other grounds than those specified in terms of need and prognosis, a recent example being the UK cancer fund.^2 3^ ^3^

In moral philosophy and ethics, debates are ongoing on how this set of principles may be justified, what more exact variants appear as more viable in that light and what practical conclusions may in fact be deduced from such theoretical explorations.^4 5^ At this level, there are, of course, long-standing disagreements between utilitarian-oriented consequentialists, egalitarians, desert theorists, Kantians, and so on. In this paper we will, however, for the most part keep these debates in the background. We will, however, return to them in the fifth section, when exploring the prospect of justifying our suggestion of adding a sustainability principle to the set of operational principles.

**Figure 1:**
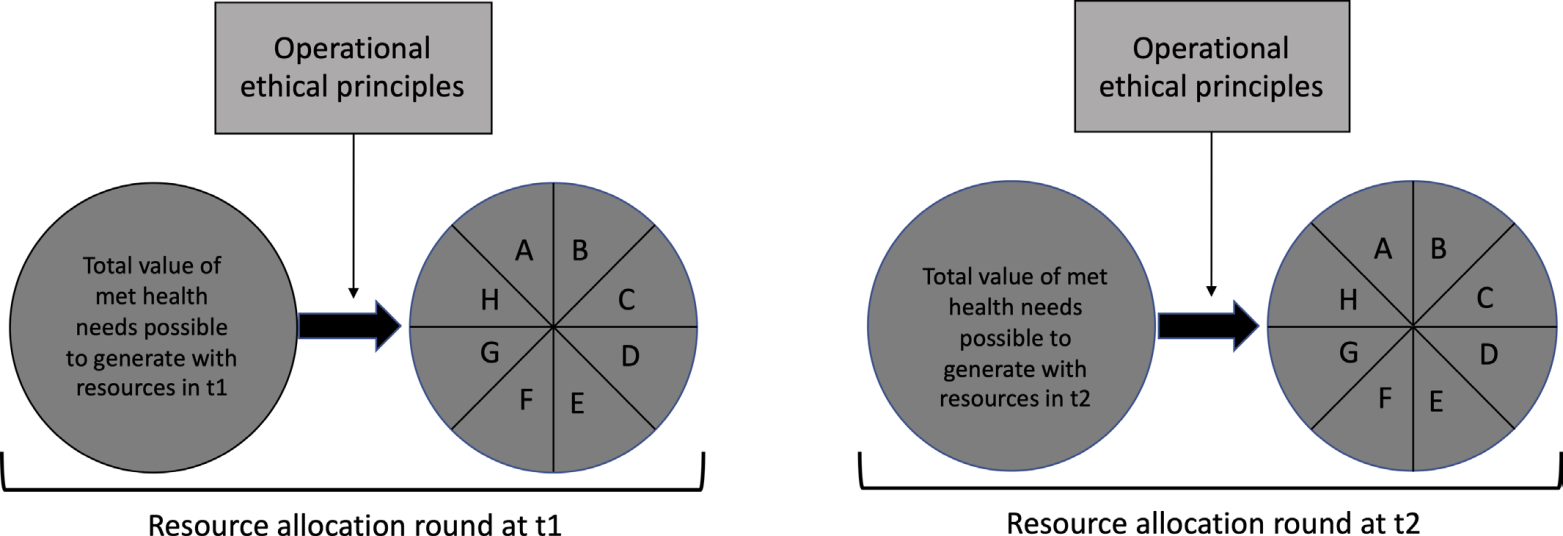
The temporal structure of operational healthcare resource allocation.

The justification of healthcare resource allocation decisions thus makes use of (some variant of) the set of operational principles above. However, this set is also used in a particular way, determined by the way that new resources in practice become available within a health system. Typically, this follows some variant of a fiscal year pattern, where resource allocation decisions are made relative to a certain, time-limited ‘pie’ of resources that needs to be ‘sliced’ in appropriate pieces (to be handed to appropriate receiving parties). The set of principles above is in practice used to justify this particular slicing pattern—if it conforms to the principles it is justified. This holds regardless of what particular variant of the set is applied (regarding interpretation of the principles, and determination of their role and relative weight). The justification holds until it is time for a new pie to be sliced (eg, the next fiscal year). Thus, besides the operational principles, the actual practice of justifying resource allocation in healthcare also includes a temporal structure, illustrated in figure 1.

## The sustainability problem of (justified) healthcare resource allocation

From a standard healthcare ethical standpoint, the question of what is a justified resource allocation will be answered once a ‘pie’ of resources has been ‘sliced’ according to a pattern supported by the operational principles. However, this notion of justified resource allocation is complicated by a phenomenon that we will call *negative dynamics*. Negative dynamics enter the reality of justifying healthcare resource allocation due to its temporal structure. Since decision-makers are never in a position to evaluate how to allocate the sum total of available resources across time, but only ‘time sliced pies’, a particular allocation decision may impact on the conditions for future allocation decisions in ethically relevant ways. Such impact may be both positive and negative.

To present this problem succinctly, it is useful to specify the ethically relevant ‘currency’ in which the justification of healthcare resources should be formulated. Clearly, the ethically relevant way of conceiving what makes up the ‘pie’ to be ‘sliced’ is not the amount of money, time, infrastructure or something like that (although this is what is being allocated in practice). What needs justification is the distribution of value achieved by spending money, time or use of infrastructure in one way rather than another. If no such value resulted, the use of the resource would be obviously unjustified, as would the very enterprise of healthcare. The type of value concerned is, in turn, indicated by the operational principles. More positive value results when more reduction of more severe needs on equal conditions results from a distribution pattern. Therefore, henceforth, when we speak about justifying the ‘slicing’ of a ‘pie’ and the ‘size’ of this pie, this should be understood in terms of the value possible to generate by a round of resource allocation. Based on this notion, let us now present the phenomenon of resource allocation dynamics more closely.

A well-known positive example of such dynamics is when healthcare resources are allocated so that a decrease of the future need of healthcare results. This is the typical idea behind vaccination programmes, where the main benefit takes the form of future absence of disease in the population. Due to the investment in a successful vaccination programme, there will be more healthcare resources available per healthcare need in the future, and these needs can be better met than if they had been forced to ‘compete’ with the healthcare needs avoided due to the programme. Another example is when the overall effects of healthcare boost the future income of the healthcare system, so that more resources become available in the future than what would otherwise have been the case, for example, by enabling people who would otherwise have been on sick leave to return to work and pay income tax. It is common to appeal to such dynamics to help justify allocation of resources by pointing to costs of an intervention being offset by future cost reductions or income increases for healthcare.^4^

**Figure 2:**
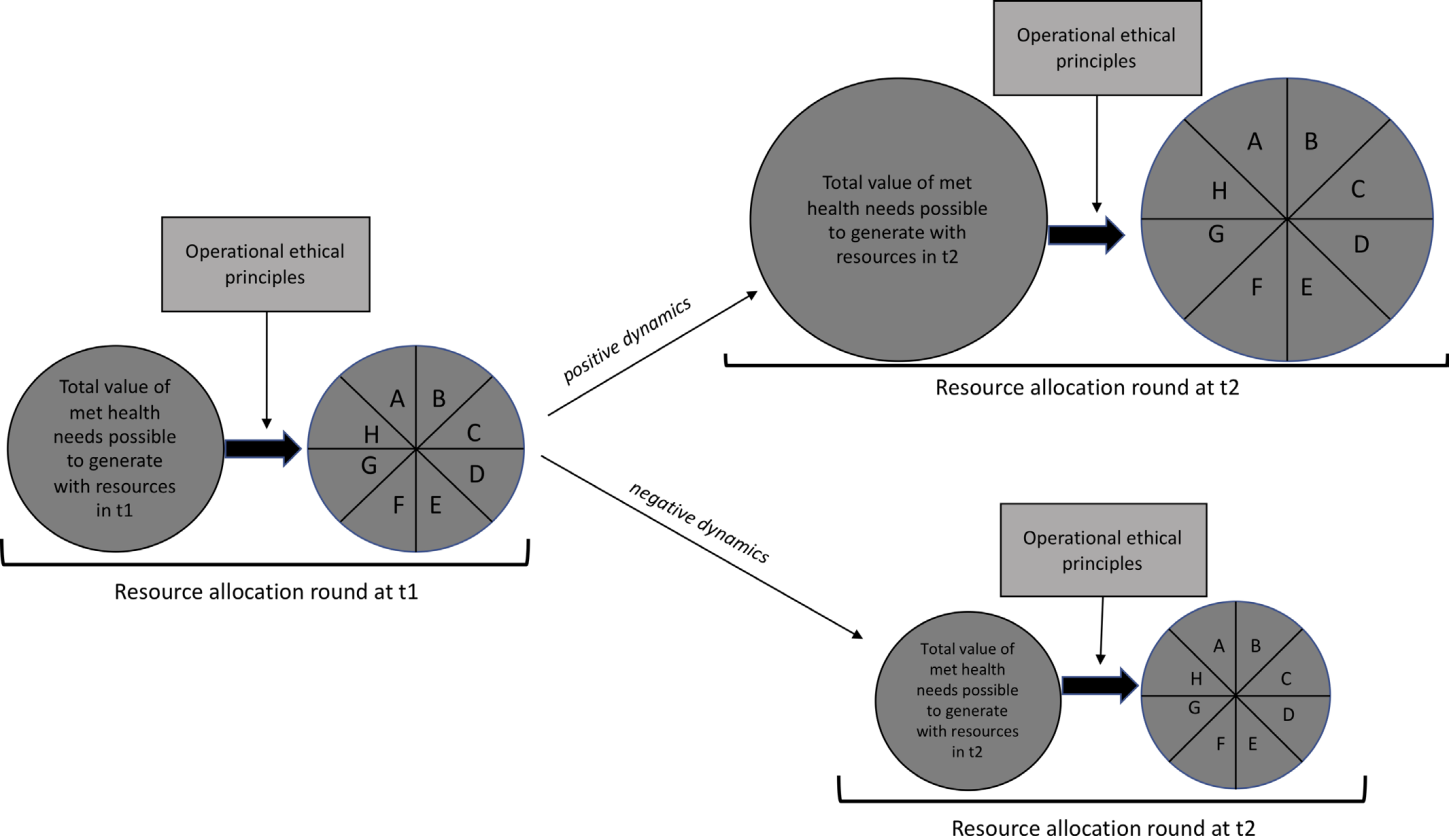
Positive and negative temporal dynamics of operational healthcare resource allocation.

Both of these are examples of *positive* dynamics of a resource allocation: more resources per health need become available for future allocations. There are also other examples than these two, but the defining characteristic here is that a resource allocation according to the operational principles positively affects the available outcome value possible to generate through a future resource allocation that uses these principles. *Negative* dynamics, in contrast, occur when the opposite ensues between two temporally separated resource allocation rounds. That is, a justified (according to the operational principles) resource allocation has the effect that less value can be generated through a future justified resource allocation. The structure of both the positive and the negative dynamics is illustrated in figure 2, where the size of the ‘pie’ sliced by an allocation represents the value possible to generate through this allocation.

Whereas positive dynamics are already taken into account in present applications of the operational principles, as indicated above, negative dynamics are not. We provide three examples that illustrate this omission. The first concerns a presently widely discussed development in the expected health effect of new drugs related to cost per health effect unit of these drugs (however that is measured; eg, quality-adjusted life-year). An increasing number of new drugs targeting very serious conditions have very modest or very uncertain clinical effects (sometimes only effects on biomedical markers of uncertain clinical relevance) but are nevertheless listed at high, sometimes very high, prices.^6 7^ Many healthcare systems use the operational principles to prioritise these drugs due to the severity of the condition, and allocate resources for buying them in spite of the weak prognosis. As a result, when these resources are spent, much less healthcare need is met than if the resources had been allocated to drugs with better or more certain effects.^3 8^ This ‘negative balance’ of unmet healthcare need that could have been met with another resource allocation is transferred into the next round, and generates a larger total of unmet healthcare needs for which to allocate resources. If the amount of resources available remains the same, this means that the ‘pie’ of value possible to generate through this allocation shrinks: there are less resources available per unit of healthcare need. In the next round, this effect will accumulate, resulting in an even smaller pie of value to generate in the following round—and so on. Of course, this negative dynamic may be initially neutralised by increasing the amount of available resources at each temporal stage. However, these resources have to be taken from somewhere else, and if the dynamic continues, this response will become impossible (to justify) at some point. Negative dynamics thereby continuously undermine the economic basis of the healthcare system, as more and more resources are allocated to generate less and less satisfaction of healthcare need. At some point, the economic basis of the healthcare system will collapse, and hardly generate any value, in spite of costing enormous amounts of money. The basic problem here is a lack of economic sustainability of the justified resource allocations resulting from application of the operational principles.

Another example of negative dynamics comes from the fact that the production of antibiotics (which are essential for all modern healthcare systems) has been found to emit vast amounts of resistance-driving residue.^9^ This pollution is contributing to the massive global health challenge of antibiotic resistance,^10 11^ the backbone of which can be described in terms of a drastic reduction of healthcare needs that can be met by the system. Growing antibiotic resistance thereby systematically reduces the size of the ‘pie’ of value available for allocation in the future, and this process is fuelled by the way in which antibiotics are produced. Producers have no reason to change these production practices, however, as procurement of the drugs they produce (based on the operational principles for healthcare resource allocation) is made independently of any environmental externality. As healthcare systems find reason to prioritise clinically equivalent drugs that cost less, a producer will generate a better yield through offering a lower price for an equivalent (antibiotics) product by cheaper production practices that allow pollution. Thus, in this situation, the operational principles will favour a cheaper equivalent drug regardless of their gradual contribution to the antibiotic resistance problem, as this will generate more healthcare need fulfilment per available resource unit in this round. Likewise, they will disfavour any producer that improves the environmental aspects of production and compensates for the cost with a higher price. For each round of justified resource allocation on this premise, resources will be channelled into the hands of producers that contribute to the antibiotic resistance problem, and the future accumulated effect is that this problem increases, thus gradually undermining the effectiveness of healthcare, and shrinking the potential for meeting future healthcare needs. The basic problem here is a lack of environmental (health) sustainability of the justified resource allocations resulting from application of the operational principles.

A recent contribution^12^ describes how high-income healthcare systems could act to respond to antibiotics pollution by taking environmental considerations into account in the resource allocation linked to antibiotics procurement. Such a move brings some ethical challenges, and some delicate questions of the exact design of the relevant incentive structures, but a strong case in favour of this kind of solution can be mounted^13^ and actions of this sort have recently been jointly advocated by leading global organisations in the area.^14^ However, the operational principles in existing healthcare resource allocation systems do not support such considerations, as the immediate effect in the present round (eg, a fiscal year) would be that less healthcare need can be met. The upside of taking environmental production externalities into account, in terms of preventing impaired ability to meet healthcare need due to increased antibiotic resistance, would materialise in the future, not in the present round of resource allocation. The situation thus resembles the shrinking economic room for meeting healthcare needs in the first example: the relevant effect resides in the future, and is not a health effect in the present round of allocation.

A third example of negative dynamics is related to drug shortages, which is a persistent problem in both developed and developing societies and the object of growing attention from governments, scholars and non-governmental organisations.^15–17^ A shortage means that a drug becomes unavailable, implying that patients may have to resort to a less effective, more risky and/or more expensive treatment, or see their treatment need go unfulfilled.^15^ Antibiotics are particularly affected due to fragmented supply chains and low profit margins, and shortages in this area are especially disconcerting since they compound the antibiotic resistance problem.^16^ Though the causes of shortages are complex, at bottom there is a lack of incentive for drug manufacturers to ensure supply reliability.^17^ Since the market does not reward supply reliability above the minimum level demanded by regulators and procurers, companies do not invest more than needed in this light.^16^ By systematically procuring and subsidising the cheapest among interchangeable drugs without strict expectations related to supply reliability, societies effectively reward manufacturers who keep prices down by minimising spending to that effect. Here, the application of the operational principles maximises the fulfilment of health needs per unit of resources spent in the short term. But at the same time, this creates a systematically increasing risk of future shortages resulting in unmet health needs over time, which reduce the size of the ‘pie’ of available value in terms of met health needs in the long term. Since these costs arise in the future, they are not accounted for in the allocation round where the principles are applied. Hence, a similar kind of negative dynamic as in the other examples results.^5^

Sustainability is about the ability of a system or an operational pattern to continue to function over time without loss of value. An added principle of sustainability would therefore support taking into consideration the negative dynamics in each of the cases, and allow support of resource allocations that would avoid or mitigate such destructive structural effects. In the next section, we state such a principle, discuss different ways of specifying it and strengthen the case for its inclusion among the operational principles for healthcare resource allocation. In the subsequent section, we will then consider objections against including the principle.

## A sustainability principle stated and defended

As a starting point, we will formulate a generic principle of sustainability, which may be made more precise and specific in various respects:

- Sustainability: if a resource allocation pattern at time t1 produces negative dynamic effects at time t2, this to some extent counts against this pattern at t1, and in favour of resource allocation patterns at t1 with no or weaker negative dynamic effects at t2.^6^

This idea may be varied in a number of ways to be discussed below, for example, regarding strength of the reason against a resource allocation pattern producing negative dynamics, and how that reason can be related to the other operational principles. But before that, it should be noted that any such more normatively precise variant may also be discharged in different specific forms in a resource allocation system.

1. Side constraint: the principle functions like the equal treatment or cost-effectiveness principles, implying limitations to what actions could be justified by the other principles. Just as these two principles rule out allocations leading to unequal consideration of similar need and prognosis claims, or wasteful spending of resources to meet prioritised healthcare needs, the principle of sustainability rules out (excessively) unsustainable allocations.
2. Gradual weight: the principle functions as another relevant variable, besides the ones implied by the need and prognosis principles. These are all capable of producing reasons that could be balanced against the reasons produced by the other two principles to effect an all things considered judgement on what allocation should be favoured (within the limits set by the equal treatment and cost-effectiveness principles).
  In both of these specifications, the principle of sustainability is fully integrated among the operational principles. But the specific discharge of the principle may also take other forms, for instance:
3. Rational savings: rather than producing a reason for what patient groups and healthcare interventions to prioritise in the allocation round at hand, the principle here supports the administrative manoeuvre to withdraw some of the resources from this allocation round, and save them for future use to compensate for the loss of value due to negative dynamics. The principle here operates on a metalevel that relates different allocation rounds to each other and produces reasons for saving resources for the future, as well as for determining how much to save.
4. Insurance: again, this variant of the principle does not produce reasons for or against particular allocations, but rather operates across allocation rounds. In this case, the principle produces reasons for linking the resource allocation system to an insurance scheme that pays out future compensation for negative dynamics, and for paying and designing the premium.

We will in the following initially keep it undecided exactly what variant of these forms fits best to different specific kinds of allocation decisions and sustainability problems. Likewise, we will initially hold open all further normative details about the weight of the reason for sustainability, and initially assume only that there is *some* such reason.

The most obvious argument for the inclusion of a sustainability principle is that it would counteract the negative dynamics described in the previous section, avoiding or mitigating the considerable long-term costs that these generate. Such a principle would, for instance, make room for avoiding or reforming patterns of procurement, subsidy and prioritisation that are justified when assessed by the operational principles applied to each specific allocation round, but that pose a long-term economic threat to the efficiency of a healthcare system or systematically favour polluting or fragile pharmaceutical supply chains over cleaner or more secure ones.

The support for sustainability in healthcare resource allocation is furthermore grounded in normative ideals internal to the operational principles that already guide such allocation. Thus, insofar as healthcare systems attach weight to these principles, they should also adopt some version of the sustainability principle. This is perhaps most clearly seen in the case of equal treatment. This principle demands, positively, that patients who are equally situated with respect to relevant considerations (eg, need, prognosis) receive equal shares of resources and, negatively, that nobody is denied an equal share based on any *other—*arbitrary—consideration. The negative dynamics described in the previous section undermine the application of this normative ideal. This is because they generate a gradual depletion of resources available for meeting health needs and/or a gradual increase of needs to be met over time. At some point, a situation will arise where patients who at time t1 would have received some resource based on their need, prognosis, and so on, will be denied this resource at t2, since the resources available must be devoted to more serious or urgent needs. Patients at t1 and t2 will then have been treated unequally, despite being equal with respect to all relevant considerations. The only difference between them is that they happen to be affected by decisions taken at different times, but, by itself, this temporal distance is morally arbitrary.

A similar point applies to need. This principle is typically interpreted as expressing a prioritarian normative ideal: the worse off a person is, the stronger her claim on some resource that could benefit her, other things being equal.^18 19^ The negative dynamics we have described threaten the application of this principle (thus interpreted) too. When some resource is gradually depleted or the needs for that resource gradually increase, a situation will eventually arise where patients at t2 will be denied some resource available to patients at t1, even though the needs of the patients at t2 are somewhat greater than those of the patients at t1. Better off patients (ie, patients with lesser need) will then have taken precedence over worse off patients (ie, patients with greater need), in opposition to the prioritarian spirit of the need principle.

The normative ideal underlying the cost-effectiveness principle might not be vulnerable to negative dynamics in the same way. After all, cost-effectiveness primarily rests on a commitment to avoid waste: it is exclusively concerned with maximising the output generated by the use of some resource, however large or small that resource may be. The mere fact that the resource shrinks, or that claims on it grow, does not make allocations of that resource less cost-effective. However, the importance of not wasting resources is purely instrumental and relative to some idea of what resources should be used for. Societies care about cost-effectiveness not for the sake of cost-effectiveness, but because they are committed to some other value—here, the fulfilment of health needs—that can be realised in greater measure by more rather than less cost-effective allocations. If *this* concern is applied across time, it clearly seems threatened by negative dynamics, because these diminish the amount of the relevant value that can be realised.

Thus, the application of standard operational principles over time is prone to generate outcomes that undermine the normative ideals on which these very principles are based, and the sustainability principle seeks to counteract this effect. On a more abstract level, many ethical theories underlying these ideals could also support the addition of such a principle, although perhaps in different specific variants. Many such theories are future oriented, and as such they can value the dynamic effects of temporally limited resource allocations. These perspectives include (different brands of) consequentialism, harm-oriented rights theories, social contract theories of justice, and so on. Variation with regard to what variants of the sustainability principle could gain such support may regard, for instance, what determines the gravity of a case of unsustainability, or how much it counts against a resource allocation in view of the other principles. But these theories would still provide support for the general idea of a sustainability principle. That is, they could motivate *some* version of the principle in the same way as they motivate variants of the other operational principles.

Two related variations in this regard warrant mentioning. The first concerns time frame: *how far* into the future should we look when determining whether a resource allocation decision would be (sufficiently) sustainable? At first glance, answering this question may seem difficult in view of thorny issues discussed in population ethics and intergenerational justice debates concerning the justifiability of discounting the value of future events.^20^ The second concerns what *kind* of future scenarios should be taken into account (within a given time frame). Should all possible changes to the need panorama and resource situation be considered? If not, which to include and which to exclude? A possible worry here is that an overly inclusive approach would make the principle impracticable due to difficulties in predicting future outcomes.

We believe that mundane considerations on an operational level make it possible to sketch a preliminary approach to these issues, despite the theoretical complications. First, note that the sustainability principle only regards negative dynamics arising from a resource allocation decision, that is, parts of the future changed by this decision, not wholly unrelated scenarios. Second, the increasing uncertainty regarding the presence and nature of such negative dynamics the farther we gaze into the future means that, in practice, a systemic mechanism substantiating the reality of the negative dynamic needs to be demonstrated. Such mechanisms are present in all three cases presented earlier. Absent some such mechanism, the worry about negative dynamics becomes merely one of many possible scenarios for the future. This goes to show that, just as with the other operational principles, the impact of the sustainability principle, and what requirements it will issue regarding how to assess an allocation proposal, will be dependent on underlying evidence.

## Objections to sustainability in healthcare resource allocation

The support for an operative sustainability principle assumes a concern for how health needs are met in the future (beyond a particular resource allocation round) that recognises the significance of negative dynamics and the call for avoiding it. But some ethical theories are admittedly either not future oriented at all, or not future oriented in a way that would view these dynamics as morally relevant. One obvious example with clear relevance for resource allocation is desert theories of justice.^21 22^ These theories share the idea that a justified allocation of goods depends entirely on facts about the past, such as the effort people have made or their contribution to society.^23^ The future interests that the sustainability principle is meant to care for are not recognised as valid grounds for justifying any distribution of any kind of goods.

Another example is libertarian theories, which also espouse principles of justice that are purely historical, though in a different sense. Nozick^24^ argued that any distribution is legitimate as long as it has arisen through voluntary exchanges of goods that were justly acquired to begin with. On this view, the fact that there will be less goods available in the future or greater need for them has no bearing on whether or not they are legitimately distributed.

Yet another example is strict egalitarianism, according to which each person should have an equal share of whatever good(s) distributive justice is concerned with.^23^ On this view, changes to the *amount* of goods available for distribution are not relevant to evaluations of justice; a pattern of equal distribution is all that matters, independent of the long-term availability of the goods in question. Thus, the depletion of goods or the proliferation of claims on them is of no concern, as long as the goods that *are* available are divided equally among those with valid claims.

All of these ‘pure’ theories are known to face serious objections, and are often modified using elements from future-oriented theories in order to accommodate these. For instance, it is common for theorists with egalitarian sympathies to mix a concern for equal distribution with other concerns in order to avoid powerful objections to strict egalitarianism, such as the levelling down objection.^18^ Similarly, many supporters of the moral importance of desert would be willing to pay attention also to other considerations in order to avoid counterexamples, such as when a distribution of goods only according to desert would undermine social stability or basic needs, or drain future resources.^25–28^ Thus, whereas certain ‘pure’ theories may in themselves be unable to support the sustainability principle, the considerations that they are often combined with in order to increase plausibility (eg, concern for social stability or priority to the worst off) include the sort of future orientation which may support the importance of sustainable resource allocation.

At a less theoretical level, we may also note that these ‘pure’ theories sit uneasily with not only the sustainability principle, but with the already established operational principles of healthcare resource allocation, and the broader ethos supporting them. For instance, desert is either given a very limited role or is rejected altogether as basis for resource allocation in most publicly funded healthcare systems.^1^ Another, very salient example regards libertarian ideals of justice, as these famously object to the very idea of a central public agent legitimately assembling goods from individuals and redistributing these, for instance, by funding a public healthcare system.^24^ Since the issue of healthcare resource allocation addressed by the operational principles presupposes such a system, libertarians would likely reject *any* such allocation in favour of some market-based scheme (where resources are distributed according to principles of supply and demand). Thus, it seems that reigning approaches to resource allocation in publicly funded healthcare systems must, at least partly, be based on other theoretical perspectives than libertarianism or desert theory perspectives, perspectives likely to be friendlier to considerations of sustainability. The objections from desert and libertarianism challenge not so much the inclusion of a sustainability principle among the operational principles as the basic normative framework of which these principles form part. We can therefore set these objections aside for the purposes of this paper.

A possibly more damaging objection appeals to considerations internal to the established operational principles for healthcare resource allocation. We argued above that such considerations, properly understood, support the addition of a sustainability principle. However, they could also be invoked to challenge this idea. The most forceful objection of this kind is based on equal treatment. Suppose a healthcare system has to prioritise between two equally needy patient groups for whom there exist equally effective treatments. Suppose also that the treatment needed by one of the groups (but not the other) raises sustainability issues; it might, for instance, be an antibiotic the production of which is known to cause significant pollution. If sustainability considerations are included in allocation decisions, it seems that the latter group of patients would take precedence over the former, even though they are equally positioned with respect to need and prognosis. It might be argued that this violates the equal treatment principle, or the normative perspective expressed by it, since the only difference between the groups has nothing to do with facts about *them* and so does not constitute a legitimate basis for unequal treatment. Another way to put the challenge is this: including sustainability considerations in order to secure equal treatment *across* allocation rounds may objectionably sacrifice equal treatment *within* such a round.

We have three responses. First, note that the objection does not challenge all forms of the sustainability principle listed in the fourth section, but only side constraint and gradual weight. Since these introduce sustainability considerations into specific allocation decisions, they could indeed in certain cases advocate unequal treatment of groups with equally strong claims from the viewpoint of the other operational principles. However, in the forms of rational savings or insurance, sustainability considerations operate independently of such decisions and thus do not compete with standard operational principles in this way. Since these considerations come into play *before* resources are allocated to patients or interventions, they will not imply unequal allocation in a given allocation round. The objection can thus be sidestepped by having the principle take either of these two forms.

Second, even focusing on side constraint and gradual weight, the unequal treatment involved may not be of a kind that the equal treatment principle, properly understood, rejects. This principle rests on the view that just institutions should not give people unequal prospects based on factors that are, as Rawls^29^ (Section 12) puts it, ‘arbitrary from a moral point of view’. While Rawls himself had contingencies in the natural and social lotteries in mind, it remains debated just what factors are properly considered morally arbitrary. On one view, any factor outside a person’s choice or control is arbitrary.^30^ However, this cannot be the view assumed by the equal treatment principle for healthcare resource allocation, since this principle permits prioritising patients with different needs and/or prognosis differently, even though these factors are typically not within people’s control. A better suggestion (for present purposes) is the idea that whether some factor is arbitrary depends on the justification of the particular institution in question.^31^ For instance, merit is plausibly a non-arbitrary basis for unequal rewards on the labour market but an arbitrary one in the legal system because only the former institution is justified by efficiency considerations. Applying this idea to the case at hand, it seems that sustainability considerations may well qualify as non-arbitrary. Healthcare is plausibly understood to properly aim at mitigating social inequalities,^32^ or at least at meeting a population’s health needs on equal terms. Functioning in accordance with such a justification over time requires counteracting negative dynamics, for reasons already given. If unequal allocation to patient groups or interventions that other operational principles consider equal is a necessary or effective way of achieving this, then this could be seen as an instance of unequal treatment based on relevant, that is, non-arbitrary, grounds.

Third, even if these lines of reasoning fail, and unequal allocation based on sustainability remains suspect, we must not forget that *failure* to apply a sustainability principle *also* involves an objectionable form of unequal treatment. Absent such a principle, patients’ prospects will (as argued above) arbitrarily worsen over time due to negative dynamics. Thus, two egalitarian commitments, one present focused and the other future oriented, pull in different directions, and it cannot be assumed that the former should always take precedence. Rather, how to balance them in a given case remains an open question.

These responses reinforce what we have said from the start: adding a sustainability principle necessitates decisions regarding its more exact form and weight for resource allocation decision-making.^7^ In some application areas, say drug procurement by hospitals, rational savings or insurance may be easier to accept than designs that compete directly with the other operational principles *within* the same budget. Such a design would then also make salient the need to formulate how much of a sustainability threat is needed to trigger a budget adjustment. In other areas, however, allowing sustainability to immediately compete with the other operational principles (side constraint or gradual weight) may be less problematic. For instance, when society decides on subsidy for two clinically equivalent drugs, one of which poses an environmental sustainability problem, it would not disturb standard healthcare ethical thinking to have a sustainability principle motivating that subsidy should be denied to the environmentally problematic drug while granted for the other one. Again, this still leaves many questions open regarding the exact formulation of the sustainability principle, but the examples illustrate how it may be varied to fit important healthcare resource allocation decisions, and be adjusted to a wide variety of views regarding the other operational principles.

## Conclusion

We have presented a sustainability problem afflicting widely embraced models of public healthcare resource allocation, and illustrated it with regard to drug procurement costs, industrial antibiotics pollution and fragile supply chains for pharmaceuticals. We have formulated a sustainability principle that we propose should be added to standard operational principles for public healthcare resource allocation, and described different forms it may take and how its normative importance may vary. We have defended this proposal by demonstrating how it could fix the identified sustainability problem, and by appealing to normative assumptions supporting standard operational healthcare allocation principles and underlying ethical theories. We have also considered two potential objections, arguing that the first of these rests on highly controversial theoretical assumptions and is irrelevant in the present context because it rejects the basic normative framework underpinning established healthcare resource allocation regimes. However, allowing sustainability to influence healthcare resource allocation does make the ethics of this area more complex, and the second objection considered illustrates that adding a sustainability principle necessitates specifying its role, function and importance in different areas of resource allocation.

## Footnotes

The need and prognosis principles necessitate assessment of the quality of evidence supposed to demonstrate the nature of patient needs, and the expected value of interventions. This could be added as a separate principle, but for simplicity’s sake we here treat is as implicit in the stated principles. Of course, differences between the way that health systems apply and balance the operational norms may also regard what role and weight is given to the assessment of underlying evidence.

In conjunction, the need, prognosis and equal treatment principles also imply a principle that may be called ‘horizontal cost-effectiveness’, that is, a principle saying that resources should be systematically allocated to patient groups and intervention types in proportion to the reasons in terms of need and prognosis. As in the case of the need to assess evidence mentioned in the previous footnote, we treat this principle as implicitly assumed in the present context.

A major theme in the healthcare ethical discussion regards the role of desert and ‘individual responsibility’ for health when prioritising healthcare needs.^33^ Such considerations are typically absent in the operational systems that have been formulated in different countries, but there may, of course, be different background assumptions about the allocation of responsibility between health systems, professionals within them and patients when healthcare interventions are discharged and effects evaluated (eg, in terms of adherence to prescribed treatment plans and how to respond to that). While this phenomenon is surely worth more philosophical and ethical analysis,^34 35^ we here exclude it from the list of *operational* principle. We will, however, discuss considerations of this type in the fifth section, when exploring the philosophical justification of our proposed sustainability principle.

It may be asked whether or not positive dynamics should, in addition, be given a more immediately forceful role as a bona fide *health benefit* in healthcare resource allocation, at least when the dynamic is discharged via the reduction of future healthcare needs (primary prevention), and whether or not that might motivate other additions to the operational principles or not. However, this issue lies outside the scope of the present article.

In the ongoing COVID-19 pandemic, shortage of healthcare material other than drugs has been highlighted as a major resource allocation challenge.^36 37^ Insofar as these shortages are due to procurement patterns that follow the same operational principles as drug procurement, they illustrate the same kind of negative dynamics and further strengthen the case for a sustainability principle.

Note that the sustainability principle only requires accounting for negative dynamics resulting from the particular allocation pattern under consideration. The future may, of course, hold many other changes of circumstances (eg, pandemics, financial crises, governance reforms) that impact on the resources available for allocation and the pervasiveness and severity of different health needs. However, we set aside such independent changes since they do not, in our view, pose the same principled challenge to existing operational principles as negative dynamics do.

For similar observations, see ref 38 39.

## References

[1] Norheim OF Ethical priority setting for universal health coverage: challenges in deciding upon fair distribution of health services. BMC Med 2016;14:75. 10.1186/s12916-016-0624-4 27170046 PMC4864904

[2] Claxton K The UK’s Cancer Drugs Fund does more harm than good. New Scientist, 13 January 2015. Online. Available: https://www.newscientist.com/article/dn26785-the-uks-cancer-drugs-fund-does-more-harm-than-good/#ixzz6KzSLf3oY

[3] Lomas J, Claxton K, Martin S, et al Resolving the ‘cost-effective but unaffordable’ ‘paradox’: estimating the health opportunity costs of non-marginal budget impacts: Estimating the Health Opportunity Costs of Nonmarginal Budget Impacts. Value in Health 2018;21(3):266–75. 29566832 10.1016/j.jval.2017.10.006

[4] Bognar G, Hirose I The Ethics of Health Care Rationing: An Introduction. London, UK: Routledge, 2014.

[5] Tännsjö T Setting Health-Care Priorities: What Ethical Theories Tell Us. Oxford, UK: Oxford University Press, 2019.

[6] Kovic B, Jin X, Kennedy SA, et al Evaluating progression-free survival as a surrogate outcome for health-related quality of life in oncology: a systematic review and quantitative analysis. JAMA Intern Med 2018;178(12):1586–96. 10.1001/jamainternmed.2018.4710 30285081 PMC6583599

[7] Rodriguez-Monguio R, Spargo T, Seoane-Vazquez E Ethical imperatives of timely access to orphan drugs: is possible to reconcile economic incentives and patients' health needs? Orphanet J Rare Dis 2017;12(1):1. 10.1186/s13023-016-0551-7 28057032 PMC5217554

[8] Juth N For the sake of justice: should we prioritize rare diseases? Health Care Anal 2017;25(1):1–20. 10.1007/s10728-014-0284-5 25145639

[9] Larsson DGJ Antibiotics in the environment. Ups J Med Sci 2014;119(2):108–12. 10.3109/03009734.2014.896438 24646081 PMC4034546

[10] Larsson DGJ, Andremont A, Bengtsson-Palme J, et al Critical knowledge gaps and research needs related to the environmental dimensions of antibiotic resistance. Environ Int 2018;117:132–8. 10.1016/j.envint.2018.04.041 29747082

[11] Laxminarayan R, Duse A, Wattal C, et al Antibiotic resistance—the need for global solutions. Lancet Infect Dis 2013;13(12):1057–98. 10.1016/S1473-3099(13)70318-9 24252483

[12] Nijsingh N, Munthe C, Larsson DGJ Managing pollution from antibiotics manufacturing: charting actors, incentives and disincentives. Environ Health 2019;18(1):95. 10.1186/s12940-019-0531-1 31694717 PMC6833301

[13] Malmqvist E, Munthe C What high-income states should do to address industrial antibiotic pollution. Public Health Ethics 2020 10.1093/phe/phaa020 PMC7765630 33391392

[14] World Health Organization (WHO), Food and Agriculture Organization (FAO), World Organization for Animal Health (OIE) Technical brief on water, sanitation, hygiene (WASH) and wastewater management to prevent infections and reduce the spread of antimicrobial resistance (AMR. Geneva: WHO, FAO & OIE, 2020 https://www.who.int/water_sanitation_health/publications/wash-wastewater-management-to-prevent-infections-and-reduce-amr/en/

[15] De Weerdt E, Simoens S, Casteels M, et al Clinical, economic and policy implications of drug shortages in the European Union. Appl Health Econ Health Policy 2017;15(4):441–5. 10.1007/s40258-016-0264-z 27480539

[16] Access to Medicine Foundation (AMF) Shortages, Stockout and scarcity: the issues facing the security of antibiotic supply and the role for pharmaceutical companies. Amsterdam, NL: AMF, 2018 https://accesstomedicinefoundation.org/publications/shortages-stockouts-and-scarcity-the-issues-facing-the-security-of-antibiotic-supply-and-the-role-for-pharmaceutical-companies

[17] Food and Drug Administration (FDA) Drug shortages: root causes and potential solutions. Silver Spring, MD: FDA, 2019 https://www.fda.gov/drugs/drug-shortages/report-drug-shortages-root-causes-and-potential-solutions

[18] Parfit D Equality and priority. Ratio 1997;10(3):202–21. 10.1111/1467-9329.00041

[19] Gustavsson E, Juth N Principles of need and the aggregation thesis. Health Care Anal 2019;27(2):77–92. 10.1007/s10728-017-0346-6 28866792

[20] Meyer L Intergenerational Justice : Zalta EN, The Stanford encyclopedia of philosophy. Stanford, CA: Stanford University, 2020 https://plato.stanford.edu/entries/justice-intergenerational/

[21] Feinberg J Doing and deserving: essays in the theory of responsibility. Princeton University Press: Princeton, NJ, 1970.

[22] Lamont J Incentive income, Deserved income and economic Rents. J Polit Philos 1997;5(1):26–46. 10.1111/1467-9760.00022

[23] Lamont J, Favor C Distributive Justice : Zalta EN, The Stanford encyclopedia of philosophy. Stanford, CA: Stanford University, 2017 https://plato.stanford.edu/archives/win2017/entries/justice-distributive/

[24] Nozick R Anarchy, State, and Utopia. New York: Basic Books, 1974.

[25] Sen A Equality of What? : The Tanner Lecture on human values, I. Cambridge: Cambridge University Press, 1980: 197–220.

[26] Miller D Market, state, and community. Oxford: Clarendon Press, 1989.

[27] Milne H Desert, effort and equality. Journal of Applied Philosophy 1986;3:235–43. 10.1111/j.1468-5930.1986.tb00423.x

[28] Steiner H, Vallentyne P Libertarian Theories of Intergenerational Justice : Gosseries A, Meyer LH, Intergenerational justice. Oxford: Oxford University Press, 2009: 50–76.

[29] Rawls J A Theory of Justice. Harvard, MA: Harvard University Press, 1971.

[30] Knight C Luck Egalitarianism. Philos Compass 2013;8(10):924–34. 10.1111/phc3.12077

[31] Scanlon TM Why does inequality matter? Oxford: Oxford University Press, 2018.

[32] Daniels N Just Health: Meeting Health Needs Fairly. New York: Cambridge University Press, 2008.

[33] Sandman L, Gustavsson E, Munthe C Individual responsibility as ground for priority setting in shared decision-making. J Med Ethics 2016;42(10):653–8. 10.1136/medethics-2015-103285 27495235

[34] Brown RCH, Savulescu J Responsibility in healthcare across time and agents. J Med Ethics 2019;45(10):636–44. 10.1136/medethics-2019-105382 31221764 PMC6855791

[35] Björk J, Helgesson G, Juth N Better in theory than in practise? challenges when applying the luck egalitarian ethos in health care policy. Med Health Care Philos 2020;23(4):735–42. 10.1007/s11019-020-09962-3 32566983 PMC7538444

[36] Emanuel EJ, Persad G, Upshur R, et al Fair allocation of scarce medical resources in the time of Covid-19. N Engl J Med 2020;382(21):2049–55. 10.1056/NEJMsb2005114 32202722

[37] Ranney ML, Griffeth V, Jha AK Critical Supply Shortages - The Need for Ventilators and Personal Protective Equipment during the Covid-19 Pandemic. N Engl J Med 2020;382(18):e41. 10.1056/NEJMp2006141 32212516

[38] De Grandis G Pharmacogenomic inequalities: strategies for justice in biomedical research and healthcare. Diametros 2017;51:153–72.

[39] Venkatapuram S Health justice. Cambridge, UK: Polity Press, 2011.

